# Scoping Review of frequently highlighted attributes of Medical Professionalism in an Undergraduate Medical Education Context

**DOI:** 10.12669/pjms.37.4.4004

**Published:** 2021

**Authors:** Kamran Sattar, Muhamad Saiful Bahri Yusoff, Wan Nor Arifin, Mohd Azhar Mohd Yasin, Mohd Zarawi Mat Nor

**Affiliations:** 1 Kamran Sattar, MBBS AcadMEd AoME (UK), MMedEd UoD (UK), PhD Scholar. Department of Medical Education, School of Medical Sciences, Universiti Sains Malaysia, Kelantan, Malaysia; 2 Muhamad Saiful Bahri Yusoff, MD, MSc, PhD. Department of Medical Education, School of Medical Sciences, Universiti Sains Malaysia, Kelantan, Malaysia; 3 Wan Nor Arifin: MBBS, MSc. Biostatistics and Research Methodology Unit, School of Medical Sciences, Universiti Sains Malaysia, Kelantan, Malaysia; 4 Mohd Azhar Mohd Yasin: MD, MMed (Psy). Department of Psychiatry, School of Medical Sciences, Universiti Sains Malaysia, Health Campus, Kelantan, Malaysia; 5 Mohd Zarawi Mat Nor: PhD. Department of Medical Education, School of Medical Sciences, Universiti Sains Malaysia, Kelantan, Malaysia

**Keywords:** Medical professionalism, Medical students, Undergraduate, Medical education, Frequently highlighted, Professionalism attributes

## Abstract

**Background and Objectives:**

Medical Professionalism (MP) establishes the trust between society and doctors. We aimed at finding frequently highlighted qualities of MP in the literature.

**Methods:**

We searched PubMed and Scopus for attributes of MP, using terms, “Professionalism,” “Medical Students,” and “Undergraduate Medical Education”. We included English language, original research articles with MP attributes from the perspective of undergraduate medical education, any nationality, race, gender, and age range, as the central topic of the article. Papers published from January 1^st^ 1986 to 29^th^ February 2020 were included.

**Results:**

From 1349 identified articles, finally, 18 were included, authored in 10 countries, collectively contributing to answering the scoping review question. Two themes were identified: **(1)** *Nurturing of MP*, 11 (61.11%) out of 18 included articles, highlighted “*respect*” as the most dominant attribute as it appeared in 6 (54.55%) out of 11 reviews, “*communication*” 5 (45.45 %) studies and “*honesty*” and “*integrity*” 4 (36.36%). **(2)** *Assessment of MP*, 7 (38.89%) studies, and majority, 4 (57.14 %) assessed MP using American Board of Internal Medicine’s elements of MP, viz, “*altruism, accountability, excellence, duty, honor and integrity, respect for others*.”

**Conclusions:**

Themes exemplified MP’s most discoursed issues. The attributes are frequently used worldwide. MP deliberates as a commitment toward the individual patient, society, and necessitates transforming from its present generic form to more explicit details.

## INTRODUCTION

For many years, society and physicians have used the word *professional* to designate medical practitioners. Recently, the importance of medical professionalism (MP) has been acknowledged as a core element of medical education as well as a social contract between doctors and society.^1^ Although in almost every society—past and present— medical professionals have been expected to exhibit professional behavior, yet, MP has not been explicitly taught. This might be because of the challenges posed by the various definitions of MP. However, the trend is changing, and in recent times, it is believed that MP is not a notion that captivates medical students; therefore, there is an increased emphasis on the need to teach this subject more explicitly.^2^

The principles of MP are already well-established. They comprise selflessness, always putting patients’ interests first, focusing on a course much greater than oneself, committing to take the high responsibility to deliver good quality care, and the expectation that doctors should be accountable to not only their patients but also to the population at large. MP’s doctrine must be taught to the next generation of doctors to build an ethical foundation that allows these professionals to attain balance, have empathy, and to be caring, tolerant, and kind. Even with the shortcomings, it is evident that MP has risen as an increasingly popular subject and an indispensable part of medical education as well as a practice worldwide.^3^ Moreover, the researchers have started to explore MP and its origin. As a result of this pursuit, the attributes of MP are being explored, a myriad of definitions are being developed, and methods of teaching and assessing MP are being devised.

We see an ever expanding emphasis on professionalism in the philosophy of medical education. There have been many endeavors to define MP, yet none have gained a global settlement. Unfortunately, to date, no clear definition is resolved, let alone teaching or creating assessment methods.^4^

Undergraduate medical education revolves around medicine and surgery as main topics; therefore, these are professions demanding professionalism as an entity of primary significance within its vital stakeholders, precisely, the medical students. One question to consider is, how do we, as academics, view and define MP, and how do we convey these values to students? The school of thought is that it is imparted by thorough guidance, mentoring, and nurturing, mostly by senior faculty. Still, without defining MP and listing its consensus agreed attributes, its training and measuring remain very challenging. Therefore, it is virtually unavoidable to come to a list of unanimously agreed attributes of MP, which could build a consensus across all stakeholders even from various parts of the globe. This shall pave the path to convey MP’s true meaning to all the participants.

This should enable medical educators to develop the curriculum, plan the teaching-learning and assessment, as well as the ongoing professional development of educators and practitioners. Therefore, this review is carried out to identify attributes of MP, which are essential and frequently highlighted in the literature across the globe.

Our study aimed to explore a categorical understanding of the current literature base adjoining common highlighted attributes of MP in the undergraduate medical education context. The research objectives of this review are as follows:

1. Explore the contemporary literature to identify frequently highlighted attributes of MP.
2. Summarize the essential attributes of MP represented within literature reports across the globe.

## METHODS

In line with the aims of this study, the authors selected the approach of scoping review, which has widespread use for synthesizing research evidence. This technique offers an organized approach to assemble contextual information. We followed the framework of scoping review developed by Arksey and O’Malley^5^ and carried out the following five steps: 1) identify the research question, 2) identify relevant studies, 3) study selection, 4) chart the data, and 5) collate, summarize, and report the results.

### Identifying the research question

The research question for our study was: What are frequently highlighted and the essential attributes of MP in an undergraduate medical education context?

### Identifying relevant studies

To ensure a comprehensive search, our strategy aimed to locate both published and unpublished literature. A three-step search approach was used in each section of this review. First, a primary search through PubMed and Scopus was commenced in February 2020, and the authors carried out an analysis of the text within the title and abstract and of the index terms used to designate related articles. Second, a search using all the recognized keywords and index terms was undertaken across all databases indicated. Third, the reference list of all involved studies was searched for additional studies. To avoid missing any relevant research, broadly defined heading terms were used in the search strategy, and the electronic search strategy was carried out in collaboration with a librarian. Initial keywords in the search terms included: “Professionalism,” “Medical Students,” and “Undergraduate Medical Education.” Furthermore, reference lists in key articles were also searched for relevant articles that could have been missed during the initial search. We also searched through existing networks, related organizations, and conferences for pertinent available items, including unpublished papers. The search for unpublished studies and gray literature was carried out through Google Scholar. After this secondary search via the search engines mentioned above, the subsequent articles that met our inclusion criteria were included for further workup.

### Study selection

An extensive database search was achieved considering prior piloted and specific inclusion-exclusion criteria, which was established on focus areas identified according to our research question. Our inclusion criteria consisted of a *date range:* from 1^st^ January 1986 to 29 February 2020, *type of article:* original publications (full texts, English) with an overwhelming theme as professionalism, *participants*: medical undergraduate students and faculty.

**Fig.1:**
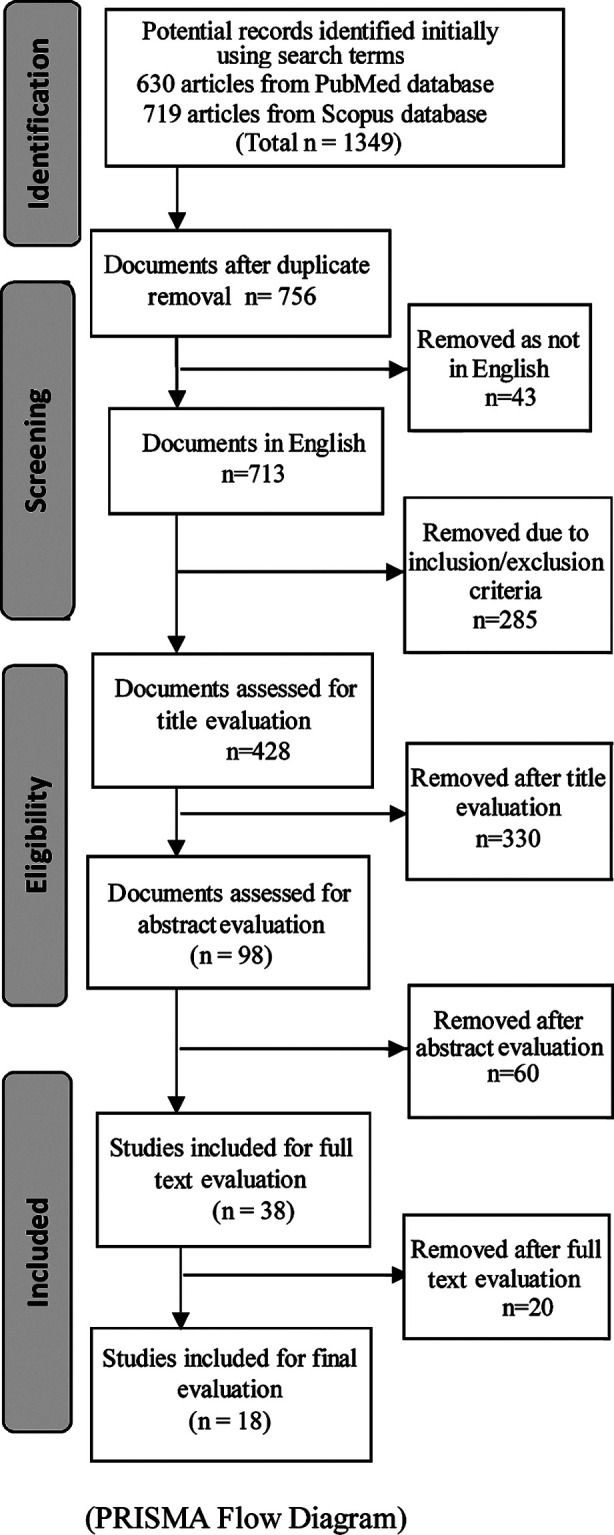
The scoping review consort diagram describing articles’ selection for the medical professionalism review.

EndNote X9 bibliographic manager was used for the citations compilation and duplicate removal. Two reviewers (KS, MSBY) independently carried out the screening for titles and abstracts. Titles and article abstracts were further constrained for relevance with our searched terms. Moreover, the full texts of the articles were examined to conclude appropriateness for inclusion in the review. The authors considered studies that included attributes of MP during undergraduate medical education (irrespective of nationality, race, gender, age). An initial search yielded 1349 documents. The documents, not consistent with inclusion criteria were removed. After a stepwise review of titles, abstracts, further removal happened, leaving behind smaller number. In the final step, full texts of the articles were evaluated, and 18 studies were finally selected (Fig.1).

### Charting the data

Ten randomly selected articles were reviewed individually by two researchers who worked toward enlisting abstraction form for the data independently, ensuring the process was consistent with the research question. Additionally, once they finished charting data from the ten studies, a discussion to decide whether their approach to data extraction was consistent with the research question and the purpose carried out. Such a step added value to our data acquisition by providing us with a pilot information about the data collection approach to reach an agreement and to include additional classifications pertinent to our research probe. It enriched the quality and dependability of the chart used. The ultimate form comprised study characteristics (e.g., author, year of publication) and outcomes (e.g., findings relevant to the research question). We had three phases during the coding process: open coding, creating categories, and abstraction. We employed an additional step by performing open coding using two investigators who, independently, described the main research areas for each article after reading the notes and headings. A list of initial codes was generated after recording the heading and notes. Subcategories from similar codes were achieved through a cyclical process. Both reviewers had a conversation regarding any disagreements and agreed on the best final explanation. The principal investigator charted all 18 articles. This safeguarded inter-reviewer reliability for the complete process and added the uniformity of our gathered data. During this whole process, regular communication was retained for a clear framework and stable charting process. The detail of the extracts of the included articles of the study is displayed in Table-I.

**Table-I:** List of original MP articles included as the selected studies for final evaluation with the retrieved information.

(Author /Year)	Location	Aims/objective/purpose	Sample/ study design/instrument	Important results and specific information related to the review question
Lazarus, Chauvin, Rodenhauser, Whitlock 6 (2000)	USA	“To evaluate the program for Professional Values and Ethics in Medical Education (PPVEME) to return the focus of our curriculum to the physician-patient–community relationship and to the nurturing of professionalism.”	Students, residents, and faculty. Surveys, focus groups, and portfolios	PPVEME is an endeavor to incorporate professionalism within the learning environment. PPVEME is built around the following themes: “Integrity, communication, teamwork, leadership, and service.”
Ber, Alroy 7 (2002)	Israel	“To promote an institutional environment/atmosphere/culture of professional behavior.”	7-10 student. Experimental/Observation Trigger films, discussions & role play.	Trigger films used were based on various themes, including: “Care, patients’ dignity and privacy, respect, up to date knowledge and skills, identifying the limits, being honest and trustworthy, confidentiality, making sure that your personal beliefs do not prejudice your patients’ care, and working in a team.”
Robins, Braddock Iii, Fryer-Edwards 19 (2002)	USA	“To examine the practicality of using the taxonomy of American Board of Internal Medicine’s (ABIM’s) Project Professionalism.”^18^	114 students. Survey with open-ended questions.	ABIM’s 18 taxonomy used in this article with the following elements: “Altruism, accountability, excellence, duty, honor, and integrity; respect for others.”
Bryan, Krych, Carmichael, Viggiano, Pawlina 8 (2005)	USA	“To determine if peer evaluation and self-evalu8ation used in conjunction and implemented early in the medical curriculum can serve as useful tools to assess and provide feedback regarding professional behavior in first-year medical students.”	213 students. Questionnaire, self and Peer evaluation	Students were deficient in professionalism evaluations. The majority of written comments from the evaluation of professionalism were related to: “Inter-professional respect, responsibility, and excellence.”
Jha, Bekker, Duffy, Roberts 9 (2006)	UK	“To describe the views and experiences individuals have about MP.”	23 students, doctors, allied health professionals, and lay professionals. Cross-sectional survey design Qualitative Semi-structured interview.	Exploration was done for: (1) “Conceptual example (honest, trustworthy, competent)”; (2) “Behavioral example (communicating effectively, treating patients equally, working in teams).” The main themes were: “Compliance to values, patient access, the doctor-patient relationship, demeanor, professional management, personal awareness, and motivation.”
Finn, Garner, Sawdon 10 (2010)	UK	“To describes how medical students perceive professionalism and the context in which it is relevant to them.”	72 students Qualitative 13 semi-structured focus groups.	Students were alert as to what is expected. The most consistent theme was: “Context of professionalism.”
Sehiralti, Akpinar, Ersoy 17 (2010)	Turkey	“To define these characteristics, and in particular seeking the help of the students themselves to define them.”	127 students. Exploratory study Open-ended question.	Students’ perceived professionalism as being a ‘competent physician.’ The suggested attributes were about: “Interpersonal relations and communication.”
Zanetti, Keller, Mazor, Carlin, Alper, Hatem, Gammon, Pugnaire 24 (2010)	USA	“To investigate the reliability of standardized patients’ scores of students’ professionalism in performance-based examinations.”	20 students A generalizability study Observed in simulated cases	The professionalism assessment was established using: The “American Board of Internal Medicine (ABIM) 18 satisfaction scale.” “Core set of professionalism attributes.” “Trust in Physician Scale” outlined by Anderson, Dedrick.^25^”
Byszewski, Hendelman, McGuinty, Moineau 20 (2012)	Canada	“To determine student perception of professionalism.”	255 students Quantitative, Qualitative Survey.	Role modeling is the most critical component The most highly ranked behaviors were: Respect Integrity, Honesty
Al-Eraky, Chandratilake, Wajid, Donkers, Van Merriënboer 22 (2013)	Egypt & Saudi Arabia	“To develop and validate a questionnaire that measures attitudes of medical students on professionalism in the Arabian context.”	413students and interns. Learners’ Attitude of MP Scale (LAMPS).^22^	“Tangible behaviors are essential to expedite discussion, assessment, and modeling of professionalism. “ LAMPS 22 is based on ABIM’s 18 elements: “Duty, accountability, excellence, autonomy, honor, integrity, altruism, and respect.”
Gale-Grant, Gatter, Abel 26 (2013)	UK	“To explore the understanding of professionalism.”	60 third-year students. Questionnaire	Students’ view of professionalism was subjective to: “Role models, media, and parents.” The most common facets were: “Confidentiality, good medical knowledge, practical skill, promptness, hygiene, and appearance.”
Akhund, Shaikh, Ali 11 (2014)	Pakistan	“To assess attitudes of Pakistani and Pakistani heritage students about important elements of professionalism and to determine students’ preferred ways of learning professionalism.”	127 students The Penn State College of Medicine (PSCOM) Professionalism Questionnaire..	“PSCOM Professionalism Questionnaire was used, and the students rated all the attributes of professionalism as important, and there was no difference across the study years”. PSCOM maintains ABIM’s elements 18: “Accountability, altruism, duty, enrichment, equity, honor, and integrity, respect.”
Al-Abdulrazzaq, Al-Fadhli, Arshad 12 (2014)	Kuwait	“To explore the experiences and views of Kuwait final-year medical students on professionalism.”	95 students. Qualitative- Quantitative Questionnaire	The most commonly recorded attributes were: “Punctuality, respect, well-attired.” Most elements were correlated to the CanMEDS (Canadian Medical Education Directives for Specialists) labeling professionalism as; “Commitment to patients, profession, and society through ethical practice.”
Klemenc-Ketis, Vrecko 13 (2014b)	Slovenia	“To determine the views of undergraduate medical students on MP.”	179 students Qualitative Focus group	Students documented the following as the professionalism dimensions; **“**Empathy, respect, responsibility, autonomy, trust, and communication, the difference between professional and private life, teamwork, partnership and two dimensions associated with it (physician’s characteristics, external factors).”
Klemenc-Ketis, Vrecko 14 (2014a)	Slovenia	“To develop and validate a scale for the assessment of professionalism in medical students based on students’ perceptions of and attitudes towards professionalism in medicine.”	12 students Qualitative 122 students Quantitative Cross-sectional observational FGD & Delphi	“Professionalism assessment scale (PAS) can be successfully used with undergraduate medical students.” Key factors emerged: “Empathy, humanism, professional relationship, development, and responsibility.”
Bhutto, Asif, Jawaid 23 (2015)	Pakistan	“To determine the level of professionalism among undergraduate medical students at two public sector medical colleges of Karachi, Pakistan.”	494 students. Cross-sectional study Survey	The six elements of professionalism (by ABIM) 18 covered were: “Altruism, accountability, excellence, duty, honor, and integrity, and respect for others.” Elements (final year): “Accountability and Excellence.” Elements (the first year): “Altruism, Duty, and Integrity.”
Randall, Foster, Olsen, Warwick, Fernandez, Crouch 15 (2016)	Bethesda	“To describe the views of professionalism held by students and faculty.”	290 students and faculty. Qualitative Survey	Following characteristics of professionalism were yielded: “Accountability, responsibility, communication, diligence, emotional maturity, ethical (behavior), honesty, integrity, lifelong-learning patient-first, reliability, respect, service, military**** professionalism, student-teacher relationship, teamwork.”
Yadav, Jegasothy, Ramakrishnappa, Mohanraj, Senan 16 (2019)	Malaysia	“To determine the perceived unethical and unprofessional behavior among medical students.”	464 students A cross-sectional study Questionnaire	“Professionalism assessment and evaluation need to be done regularly to determine the impact on the students.” Professionalism and ethics were explored within the areas of: “Discipline, plagiarism & cheating, and sexual harassment.”

### Collating, summarizing, and reporting the results

At this stage, the authors established thematic categories of the literature, consisting of different sections, including methods, evidence, their determined outcomes, and inference drawn. Once all the data were gathered and some preliminary information had been established, the authors developed the data analysis strategy and writing of the manuscript for publication. A thematic analysis was performed to identify standard terms to define researchers’ perceptions, ideas, opinions, and views about MP. This approach resulted in more organized data, yielding two main themes: significant results and specific information related to the review question.

## RESULTS

All (1349) identified documents were reviewed partially (title and abstract) or in full, 18 were eventually counted in our review (Fig.1). The 18 articles yielded, were authored by investigators in ten countries. Of these, five studies were conducted in the United States, three in the United Kingdom, two in Pakistan, two in Slovenia and one in Israel, Turkey, Canada, Egypt and Saudi Arabia, in Kuwait and Malaysia each.

The included studies showed that little attention was devoted to exploring the most highlighted attributes of MP, but rather focused primarily on students’ perceptions of unprofessional behavior. However, in combination, they contributed to answering our research questions in the form of extracted information points connecting the two themes identified across the included studies, namely: (1) nurturing MP (2) assessment of MP. These two themes exemplify the most debated issues about MP in the undergraduate medical education context within the published literature. These themes are explained in detail below:

### Theme 1: Nurturing MP (frequently highlighted attributes)

Eleven (61.11%) out of total 18 included studies focused mainly on the theme “nurturing MP and related attributes.” Among many such attributes, the most dominant one identified was “*respect*.” This element was highlighted in six (54.55%) out of 11 studies, from which theme number-1 was derived. Next, “*communication*” was an important element, highlighted in five (45.45 %) studies. Further, two other emphasized elements were “*honesty*” and “*integrity*,” which had similar occurrences as mentioned in four (36.36%) out of 11 articles. “*Teamwork*,” was emphasized in four (36.36%) studies. Another element “*accountability*” = 3(27.27%) out of 11. Moreover, five elements, “*responsibility*,” “*confidentiality*,” “*competence*,” “*role-modeling*,” and “*trustworthy*” were accentuated in two (18.18%) studies each. Furthermore, the elements of *“communication and respect”* were highlighted (with in 4 and 6 studies consequently), i.e. ≥ 40% studies amid the “MP nurturing” theme (Table-II).

**Table-II:** MP attributes ≥ 40% highlighted within the retrieved studies.

Attributes	Frequency (%)	Themes
‘Communication’	5 (45.45 %) (out of 11 studies)	Nurturing MP
‘Respect’	6 (54.55%) (out of 11 studies)	Nurturing MP
	4 (57.14 %) (out of 7 studies)	Assessment of MP
‘Altruism, accountability, excellence, duty, honor, and integrity.’	4 (57.14 %) (out of 7 studies)	Assessment of MP

### Theme-2: Assessment of MP (frequently highlighted attributes)

Seven (38.89%) out of 18 incorporated studies focused mainly on the theme “assessment of MP” and related attributes. Studies primarily focused on establishing the most appropriate way to assess MP attributes among undergraduate medical education. Among the seven studied articles, the majority, i.e., four (57.14 %), assessed MP using ABIM’s elements of MP, which is “*altruism, accountability, excellence, duty, honor and integrity, and respect for others.”* Another element, *“responsibility”* was also assessed and used in 2 (28.57 %) studies. Moreover, *“altruism, accountability, excellence, duty, honor, and integrity”* and *“respect”* were among those that were ≥ 40% or highlighted within the retrieved articles studied amid the “MP assessment” theme (Table-II).

## DISCUSSION

The informational points extracted from the 18 selected articles produced studies mainly from a subject to thorough exploration, revealing how composite the topic (MP) itself is, in that, it comprises many constituents ranging from “a definition,” “the main attributes,” “ways in which it should be taught, and the potential tools for its assessment.” The informational points also advocate that these constituents are involved in shaping medical students’ to adopt MP in the context of undergraduate medical education. In this section, we explore the (1) implications of the critical issues identified about the MP, (2) knowledge gaps that exist on this topic, and (3) the overall limitations of the scoping review process employed.

### Implications

The articles included in the study (Table-I) highlighted essential information regarding the attributes of MP, as essential elements of teaching,^6^-^15^ hence, medical school learning exposure to these elements should impact student’s behavior positively during the later years of medical practice.^16^ Likewise, the curriculum identification of lacking attributes must be attained.^17^ Currently, MP has been a focus of three primary academic works of literature: sociology, medicine, and education. However, being a multifaceted concept, teaching, and assessment of professionalism remain a challenge, and even mutual understanding of the word professionalism is limiting. An agreed definition is essential as this should enable an exhibit of explicit and appropriate content to bring within the subject domain for teaching and assessment purposes. One of the ways to teach MP is by using specific content.^18^

Jha, Bekker, Duffy, Roberts^9^ explored conceptual professionalism (honest, trustworthy, and competent), as well as behavioral professionalism as “***communicating*** effectively treating patients equally, and ***working in teams***.” Very interestingly, similar attitudes and behaviors were also highlighted in other studies included in this scoping review. Furthermore, our scoping review found that ABIM’s elements of MP “altruism, accountability, excellence, duty, honor and integrity, respect for others” were common among the highlighted features for both themes identified. Another finding was about an element of ABIM, that is, “***respect***” occurred as the most discussed as the majority of the studies 7,12,13,15,19,20 explored it. It was also noted that, “***communication***” was the next-most frequently highlighted attribute as studies^6^,^13^,^15^,^17^ addressed it. Whereas, “***honesty***,”^7^,^15^,^20^ “***teamwork***,”^6^,^9^,^13^,^15^ “***competence***,”^17^ and “***trustworthy***”^7^ were also among the stressed attributes. Furthermore, “***integrity***” also attracted the authors for their works,^6^,^15^,^19^,^20^ whereas three studies^6^,^15^,^19^ utilized “***accountability***.”

Stakeholders are left on their own to perceive, rate, and rank MP and its lapses using available information, which is indeed inadequate. Due to lack of a strong peculiarity between professional and ethical characteristics of physicians’ demeanor, often, substantial attempts were made to incorporate MP as a component within the medical ethics curricula, yet, an extensive evaluation remains unsatisfactory. However, there remains a critical need to explicitly elucidate MP, as modeling and assessing student attitudes throughout the duration of undergraduate education would ensure self-awareness (a professionalism constituent) for demonstrating good professional character.^9^

Over the past many years, academic medicine has been dedicated to progressing MP.^21^ To build conceptual and behavioral attributes for students, providing explicit instructions for reflection and abstraction is mandatory. Because society is diverse, there is a need for more explicit instructional techniques on this topic. Therefore, it could be agreed that how health care organizations address these impending academic duties is likely to directly shape students’ experiences, perceptions, and attitudes toward attributes of professionalism.

As stated earlier, our scoping review found that among the ABIM’s 18 elements of MP, were found among the highlighted elements for both identified themes. In the following section, we detail the attributes that we found were repeatedly emphasized in our included studies and mentioned as imperative, although assessment of MP is obligatory. ABIM’s elements^18^ of MP prominently appeared in the studies.^11^,^22^-^24^ Furthermore, we noted that the attribute of “responsibility” was given a central focus in the reviews.^8^,^14^ Whereas, Yadav, Jegasothy, Ramakrishnappa, Mohanraj, Senan^16^ emphasized searching for the domains of “discipline,” “plagiarism & cheating,” and “sexual harassment” when determining dishonorable acts among medical students. Most of the included studies were found to employ many similar measurement tools (focus group, interviews, surveys, and open-ended questions), and the ABIM’s^18^ elements were addressed predominantly among the various tools used.

### Knowledge gaps

Although a substantial number of studies were acknowledged in this scoping review, most of them did not present pragmatic reasons for exploring MP with their preferred set of survey items. As a substitute, this review has discovered that most of what is known about the MP attributes are, in fact, hypothetical, idea-based, or subjective. Assessment of MP is challenging because it has no standardized definition.^4^ Still, the occurrence with which MP attributes were highlighted and explored in the review, such as respect, communication, confidentiality, and accountability, advocate for the presence of harmony about explicit aspects of MP in the undergraduate context. Further, the topic of MP requires explicit and exhaustive research to be conducted by medical educationalists and healthcare organizations alike from perspectives of finding its most highlighted attributes. Several specific knowledge gaps, as mentioned above, are evident within the two thematic areas regarding the MP identified in the scoping review. Attention needs to be given to research to aid the understanding of how best the term MP could be defined and explained. Such research could offer useful insights into what undergraduate students need to know concerning MP in any educational institute, in any community, and any country, before starting to serve in a particular society in the country or abroad.

### Limitations

A comprehensive approach was taken while scoping a range of documents to synthesize what is known about the most highlighted attributes of MP. The inclusion of steps such as having two reviewers at every level and searching for original research from more than one database supplemented the thoroughness of the procedure, and thus, serve as strengths. However, we state two main limitations; first, only English language research articles were retrieved and reviewed. Because MP has gained universal popularity, therefore, the related and relevant literature might be available in languages other than English. Second, despite the extensive search strategy and inclusion criteria, not many studies that were found and included in the review come from Middle Eastern and Southeast Asian countries. This, again, could be related to English not being the first language in those parts of the globe.

## CONCLUSIONS

This MP scoping review discovered that the understanding of MP in its very generic form might not yield sufficient positive impact on a learner’s life; in fact, it needs to be extended to the explicit details with far-reaching benefits to society. This could only be achieved if it is defined, taught, and assessed appropriately.

### Authors’ Contributions:

**KS:** has contributed to the study concept and design, data analyses and the drafting of the manuscript. **MSBY:** has contributed to the study concept, drafting and critical revisions of the manuscript. **WNA:** have contributed to data analyses. **MAMY** and **MZMN:** carried out critical revision of the manuscript. All authors approved the final version.
